# The efficacy and mechanism of vonoprazan-containing triple therapy in the eradication of *Helicobacter pylori*

**DOI:** 10.3389/fphar.2023.1143969

**Published:** 2023-05-05

**Authors:** Zinan Zhang, Fen Liu, Feiyan Ai, Xiong Chen, Rui Liu, Chao Zhang, Ning Fang, Tian Fu, Xiaoyan Wang, Anliu Tang

**Affiliations:** ^1^ Department of Gastroenterology, The Third Xiangya Hospital of Central South University, Changsha, Hunan, China; ^2^ Department of Gastroenterology, Beijing Friendship Hospital, Capital Medical University, Beijing, China; ^3^ Hunan Key Laboratory of Nonresolving Inflammation and Cancer, Central South University, Changsha, Hunan, China

**Keywords:** *Helicobacter pylori*, eradication, vonoprazan, triple therapy, efficiency, safety

## Abstract

**Purpose:** Vonoprazan (VPZ) produces a strong acid-inhibitory effect, which can potentially eradicate *Helicobacter Pylori (H. pylori)*. We aimed to assess whether a 14-day VPZ-containing triple therapy was safe and effective in the Chinese population and the potential mechanism.

**Methods:** Enrolled patients confirmed to be infected with *H. pylori* were randomly divided into four groups: VPZ + doxycycline + furazolidone, VPZ + doxycycline + amoxicillin, esomeprazole (EPZ) + bismuth + doxycycline + furazolidone, and EPZ + colloidal bismuth + doxycycline + amoxicillin for 14 days. The eradication rate, medication adherence, and incidence of adverse events (AEs) were evaluated. Inhibition of *H. pylori* by VPZ and EPZ *in vitro* was assessed. *H. pylori* treated with appropriate concentrations of VPZ and EPZ were sequenced by transcriptome analysis to explore the antibacterial mechanism.

**Results:** A higher eradication rate were observed in VPZ-containing triple therapy. No obvious differences were observed in medication adherence or the incidence of AEs. VPZ had no direct inhibitory effect on *H. pylori*, whereas EPZ directly inhibited *H. pylori* may through downregulated genes related to the ribosome.

**Conclusion:** In the Chinese population, 14-day VPZ-containing triple therapy was safe and more effective and can be used clinically as first-line *H. pylori* treatment.

**Clinical Trial Registration:** ClinicalTrials.gov, identifier NCT05097846

## Introduction

*Helicobacter pylori* (*H. pylori*) infection is one of the humans’ most common chronic infections. According to the latest epidemiological data, the overall *H. pylori* infection rate in China is 55.8% (Hooi et al., 2017). Considering China’s huge population, it may be the country with the largest number of humans infected with the bacteria. *H. pylori* is believed to be the main cause of chronic gastritis, peptic ulcer disease, gastric mucosa-associated lymphoid tissue lymphoma, and gastric cancer (Farinha and Gascoyne, 2005; Wroblewski et al., 2010; Ford et al., 2016). In 1994, the World Health Organization defined *H. pylori* as a class I carcinogen (Uemura et al., 2001). Moreover, the risk of death, complications, and recurrence of gastric cancer can be significantly reduced by eradicating *H. pylori* (Ford et al., 2020; Liou et al., 2020)*.* Therefore, effectively eradicating *H. pylori* is a crucial problem faced by clinicians.

There are several treatments for *H. pylori* eradication, including standard triple therapy (proton-pump inhibitor [PPI] + two kinds of antibiotics), bismuth quadruple therapy (PPI + bismuth + two kinds of antibiotics), and high-dose amoxicillin double treatment. Although many studies, consensus, and guidelines have provided up-to-date treatment recommendations, the overall eradication rates of *H. pylori* remain unsatisfactory (Chinese Society of Gastroenterology et al., 2017; El-Serag et al., 2018; Kato et al., 2019; O'Connor et al., 2019; Joo et al., 2020). Furthermore, the eradication rate of bismuth quadruple therapy, which is highly recommended in the Chinese guidelines, is only 79.5%–86.6% (Rokkas et al., 2021). However, according to the latest international consensus, the eradication rate must exceed 90%. Therefore, the current treatment does not meet the clinical needs of China.

The main factors affecting the eradication rate of *H. pylori* include the resistance of the bacteria to antibiotics, the medication adherence, and the effect of acid-suppressing drugs are the main factors affecting the eradication rate of *H. pylori* (Wermeille et al., 2002; McNicholl et al., 2012; Sugimoto and Yamaoka, 2018; Gu and Yang, 2020; Yan et al., 2020). *H. pylori* eradication mechanisms include inhibition of gastric acid secretion and the bactericidal effect of antibiotics. *H. pylori* enters a dormant state under acidic conditions in which external drugs do not affect *it*. At the same time, some antibiotics, such as amoxicillin and clarithromycin, easily decompose under acidic conditions. Therefore, inhibiting gastric acid secretion is key to *H. pylori* eradication.

Potassium-competitive acid blockers (P-CABs) are novel acid-suppressing drugs that competitively inhibit the binding of potassium ions to H+/K + -ATPase in gastric parietal cells. Since P-CAB, especially vonoprazan (VPZ), a representative drug of P-CAB, has a relatively high pKa value, does not require acid activation, and is not influenced by CYP2C19 pharmacogenetic polymorphism, it can achieve stronger, longer-lasting gastric acid secretion suppression (Saitoh et al., 2002; Sugimoto et al., 2006; Hori et al., 2010; Scott et al., 2015). Previous study has demonstraded that VPZ produces a stronger acid-inhibitory effect than PPIs (Kiyotoki et al., 2020). Many studies have shown that the VPZ-containing triple therapy (VPZ + two kinds of antibiotics) eradication rate is significantly higher than that of bismuth quadruple therapy (Murakami et al., 2016; Suzuki et al., 2016; Ozaki et al., 2018; Sue et al., 2018). A recent meta-analysis showed that the median eradication rate of VPZ-containing triple therapy was 91.4% (Rokkas et al., 2021). These studies indicated that VPZ has the potential to replace the conventional PPI. Especially for the clarithromycin resistant bacteria, VPZ-containing triple therapy could be a more effective treatment for *H. pylori* infection than conventional therapy (Abadi and Ierardi, 2019; Shinmura et al., 2019). Therefore, Food and Drug Administration (FDA) has approved the use of VPZ to eradicate *Helicobacter pylori* (https://www.fda.gov/). However, the treatment used in these studies only lasted 7 days, whereas Chinese guidelines require a 14-day treatment due to more serious antibiotic abuse and differences in prevalent strains in China (Hu, 2020). Unfortunately, the efficacy and safety of VPZ 14-day therapy are still lacking globally (Chinese Society of Gastroenterology et al., 2017). Only one study explore that VPZ 14-day therapy were superior to PPI 14-day therapy which were conducted in Europe and America (Chey et al., 2022). In addition, the mechanism underlying the high eradication rate of VPZ-containing triple therapy remains unknown.

In this study, we conducted a prospective single-center randomized controlled clinical trial to compare the efficacy and safety of a 14-day VPZ-containing triple therapy and a 14-day bismuth quadruple therapy. An *in vitro* experiment was also conducted to explore whether VPZ has an inhibitory effect on *H. pylori* and the potential mechanism that will provide new insights for modifying eradication treatment in the future.

## Patients and methods

This study was conducted at The Third Xiangya Hospital of Central South University from 18 June 2021, to 17 June 2022. The Ethics Committee of The Third Xiangya Hospital of Central South University (No. R21041) approved this study. The study was registered at ClinicalTrials.gov with the identifier NCT05097846.

This was an open-label study because the obvious differences among the drugs in each group. The clinicians, and patients would inevitably indicate the drugs the patients were taking which was difficult to conduct a double-blind clinical trail. The investigators responsible for the follow-up were blinded to the treatment used by the patients to reduce the bias caused by the investigators’ subjective factors.

### Patients

The inclusion criteria was as follows: Patients aged 18–80 years, male or female, with *H. pylori* infection were eligible for inclusion in the study. Diagnosing *H. pylori* infection requires at least one of the following three tests: histopathology, ^13^C urea breath test, or ^14^C urea breath test. Patients who have not received anti-*H. pylori* treatment and gave informed consent were included in the study. The exclusion criteria were: 1) allergy to any of the study drugs; 2) history of *H. pylori* eradication therapy, history of gastric surgery, pregnancy or breastfeeding; 3) lack of informed consent; 4) other problems that did not meet the requirements of this study or affected the results of the study, such as inability to communicate or follow-up. According to the latest Chinese guidelines and studies, amoxicillin (0%–5%), tetracycline (0%–5%) and furazolidone (0%–1%) were selected as the antibiotics since the lowest resistant rate. Besides, drug sensitivity test would be performed only using metronidazole and levofloxacin suggested by the Chinese guidline (Chinese Society of Gastroenterology et al., 2017; El-Serag et al., 2018; Kato et al., 2019; O'Connor et al., 2019; Joo et al., 2020). Due to the obvious side effects of tetracycline, doxycycline which has smaller side effects and similar antibacterial effects was used in this study (Wang and Wu, 2012; Zhao et al., 2021).

The enrolled patients were randomly divided into four groups: patients in the VDF-triple group received VPZ (20 mg, bid) + doxycycline (100 mg, bid) + furazolidone (100 mg, bid), VDA-triple group received VPZ + doxycycline + amoxicillin (1,000 mg, bid), EBDF-quadruple group received esomeprazole (EPZ) (20 mg, bid) + bismuth (220 mg, bid) + doxycycline + furazolidone, and EBDA-quadruple group received EPZ + bismuth + doxycycline + amoxicillin.

### Sample size

Since there was no relevant data on the 14-day VPZ-containing triple therapy eradication rate in China, we referred to the results of the previous meta-analysis with the highest quality for sample size calculation (Rokkas et al., 2021). The eradication rate of *H. pylori* was 91.4% for VPZ-containing triple therapy and 81.3% for bismuth quadruple therapy. Assuming 90% power and an alpha of 0.05 (two-sided), the PASS 15.0 (NCSS, LLC. Kaysville, Utah, United States) was used to calculate the sample size of 476 patients. We assumed that 25% of patients would be lost to follow-up; thus, 640 patients (160 patients in each group) were enrolled.

### Follow-up

**FIGURE 1:**
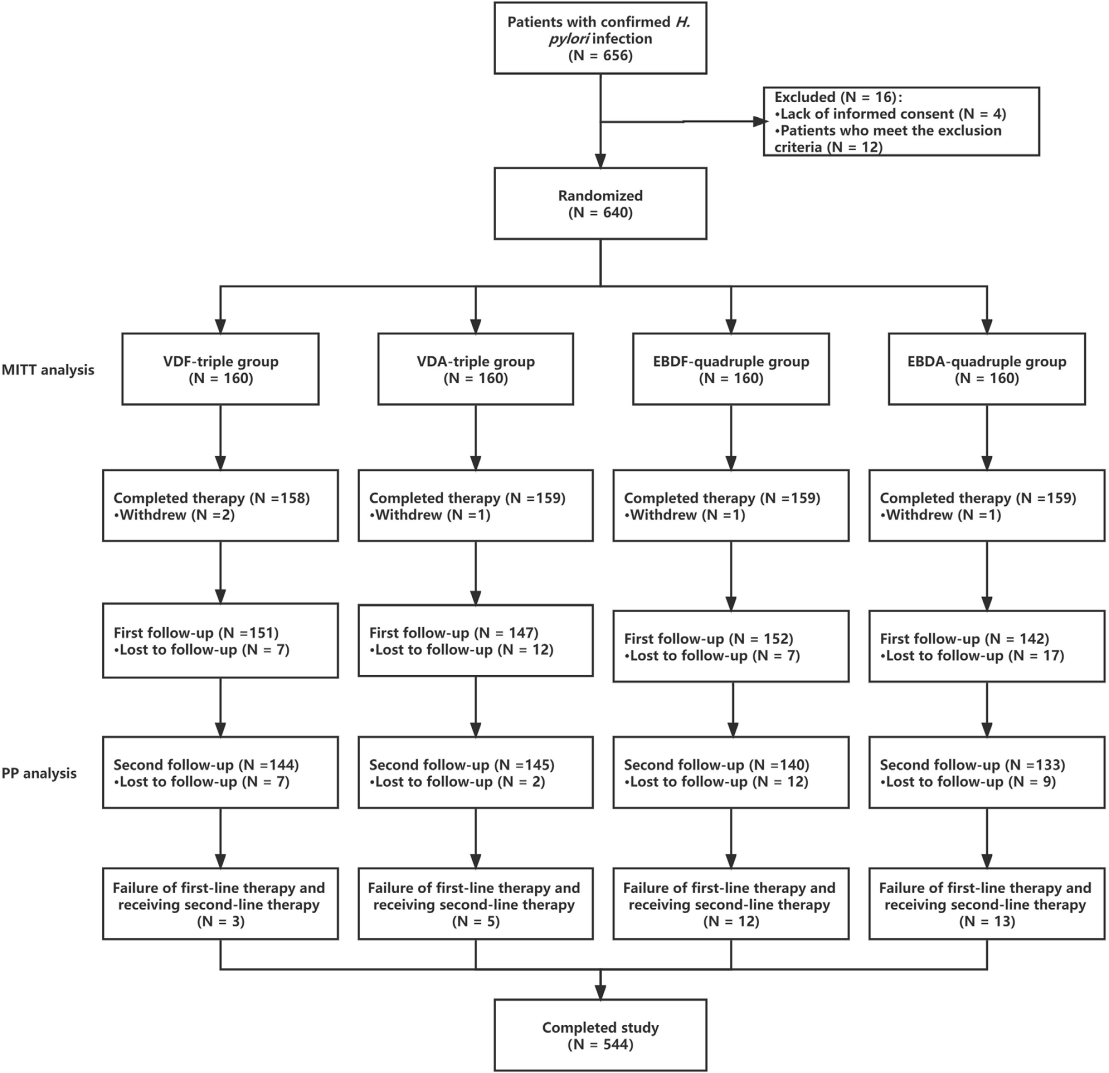
The procedure of this study.

Each patient underwent two follow-ups. The first follow-up was performed on the first day after the patients completed therapy. Medication adherence and whether relevant adverse events (AEs) occurred including whether patents had an allergy were evaluated during follow-up. Additionally, whether patients should take additional drugs, including acid-suppressing agents, was assessed. Allergic symptoms were defined as erythema, urticaria or throat edema, which gradually recover after stopping taking the drug. Acid-suppressing drugs have been used to promote healing in patients with peptic ulcers. The second follow-up was conducted 5 weeks after discontinuing PPIs, bismuth, VPZ, and antibiotics. Patients underwent a^14^C urea breath test to evaluate whether they had successfully eradicated *H. pylori* (Figure 1) during the second follow-up. For patients who failed *H. pylori* eradication, EPZ + bismuth + amoxicillin + furazolidone was used as second-line therapy.

### Outcome assessment

The primary outcome assessment was *the H. pylori* eradication rate. *The H. pylori* eradication rates for each group were analyzed using the modified intention-to-treat population (MITT) and per-protocol (PP) analyses. In the MITT analysis, patients lost to follow-up were regarded as having an unsuccessful eradication of *H. pylori*. In the PP analysis, patients lost to follow-up were regarded as dropout cases and excluded from the subsequent statistical analysis.

To comprehensively evaluate patients’ medication adherence, we used the following two methods: the Morisky Medication Adherence Scale-8 (MMAS-8) and tablet counting. The full MMAS-8 score is 8, with a score of <6, 6–<8, and 8 indicating poor, moderate, and good adherence, respectively (Morisky et al., 2008). The adherence scale was tested for reliability and validity in the Chinese population, and the overall Cronbach’s alpha coefficient was 0.83 (Si et al., 2012). For tablet counting, patients who took more than 90% of the tablets were considered to have good adherence (Georgopoulos et al., 2012).

The incidence of AEs was also evaluated. The severity of the AEs was categorized as mild, moderate, or severe. Mild referred to adverse symptoms that did not require treatment; moderate referred to patients with adverse symptoms that did not require hospitalization and could be managed only by treatment; severe referred to patients with adverse symptoms that required hospitalization before remission or that experienced study-related death. In the MITT analysis, patients lost to follow-up were considered to have AEs.

A cost-effectiveness analysis was performed. The cost mainly included the cost of diagnosis, treatment, and follow-up. The cost of drugs was determined from the Hunan Provincial Medicine Centralized Purchasing website (https://www.hnsggzy.com/). The cost-effectiveness ratio (CER) and incremental CER (ICER) are indicators of cost-effectiveness analysis. CER (yuan per percent) means the cost per 1% eradication rate. ICER means compared with another therapy, the increased cost of every increased eradication rate in one therapy.

### *H. pylori* culture and intervention

The *H. pylori* strain SS1 was used in this study. The bacteria were grown on Columbia blood agar plates implanted with antibiotics (10 mg/L vancomycin, 5 mg/L cefsulodin, 5 mg/L amphotericin B, 5 mg/L trimethoprim, and 10% sheep blood [Thermo Scientific R54008, Waltham, MA, United States)] at 37°C under microaerophilic conditions (5% O_2_, 10% CO_2_, and 85% N_2_) for 3–4 days. The bacteria were inoculated onto plates containing 20, 40, and 200 μg/mL VPZ or EPZ by the agar dilution method similar to the previous study (Saniee et al., 2016). A negative control group was set up using plates without any drugs, and a positive control group was set up using plates with 0.03 μg/mL amoxicillin. The concentration of *H. pylori* was determined by measuring its optical density at OD600 nm.

### Transcriptomic analysis

Based on the *in vitro* antibacterial test results, *H. pylori* strains treated with appropriate concentrations of VPZ and EPZ were selected for transcriptomic sequencing. Transcriptomic analysis was performed according to the method of previous studies (Chomczynski and Sacchi, 1987; Benjamini and Hochberg, 1995; Mortazavi et al., 2008), including: 1) RNA extraction, RNA library preparation, and sequencing; 2) filtering raw sequence data to obtain clean reads; 3) duplication of clean reads; and 4) identification of differentially expressed genes, gene ontology (GO) analysis, and Kyoto Encyclopedia of Genes and Genomes (KEGG) enrichment analysis. The steps are presented in Appendix 1. A *p*-value cutoff of 0.05 and a fold-change cutoff of 2 were used to judge the statistical significance of gene expression differences. The glmM gene was used as an internal reference gene in a quantitative real-time polymerase chain reaction (qRT-PCR) to verify the correctness of the transcriptomic analysis (Kansau et al., 1996).

The basic principle of the statistical method used in this study was to use the rate (%) and mean ± standard deviation to perform descriptive statistics for categorical and continuous variables, respectively. Categorical variables were compared using the chi-squared or Fisher’s exact test, and continuous variables were compared using a two-sample *t*-test. Pearson’s correlation coefficient was used to describe the correlation between the two variables. The contributions of the factors affecting *H. pylori* eradication rates were analyzed using logistic regression (conditional backward stepwise method). Reads per kilobase per million reads were used to measure gene expression. Sample size calculations were performed using PASS 15.0 (NCSS, LLC. Kaysville, Utah, United States), and statistical analyses were performed using IBM SPSS Statistics 23.0 (IBM Corporation, Armonk, NY, United States). Statistical graphs were drawn using Prism 8.0.2 (GraphPad Software, San Diego, CA, United States).

## Results

### Patient enrolment and baseline characteristics

In this study, 656 patients met the inclusion criteria, of which 16 (2.44%) refused treatment or met the exclusion criteria and were excluded from the study. A total of 640 patients were enrolled and included in the MITT analysis. Among these patients, 78 eventually withdrew from the study, including 5 (6.41%) who withdrew voluntarily, 43 (55.13%) who were lost to the first follow-up, and 30 (38.46%) who were lost to the second follow-up (or refused to be re-examined); thus, 562 patients were included in the PP analysis. No differences were observed between the groups regarding baseline patient characteristics (Table 1).

**TABLE 1:** Baseline characteristics of each group.

	VDF-triple (*N* = 160)	VDA-triple (*N* = 160)	EBDF-quadruple (*N* = 160)	EBDA-quadruple (*N* = 160)
Gender				
Male	71 (44.38%)	77 (48.12%)	68 (42.50%)	73 (45.62%)
Female	89 (55.62%)	83 (51.88%)	92 (57.50%)	87 (54.38%)
Age	42.07 ± 13.06	41.60 ± 14.89	42.44 ± 14.37	40.38 ± 13.54
Gastroscopy result				
Peptic ulcer	31 (19.38%)	32 (20.00%)	25 (15.62%)	34 (21.25%)
Chronic non-atrophic gastritis	27 (16.87%)	20 (12.50%)	31 (19.38%)	25 (15.63%)
Reflux gastritis	7 (4.37%)	13 (8.12%)	6 (3.75%)	5 (3.12%)
Atrophic gastritis	16 (10.00%)	23 (14.38%)	26 (16.25%)	20 (12.50%)
Lack of gastroscopy	79 (49.38%)	72 (45.00%)	72 (45.00%)	76 (47.50%)
Take extra medicine				
Yes	22 (13.75%)	31 (19.37%)	28 (17.50%)	27 (16.87%)
No	138 (86.25%)	129 (80.63%)	132 (82.50%)	133 (83.13%)

Note: VDF-triple, VPZ + Furazolidone + Doxycycline; VDA-triple, VPZ + Amoxicillin + Doxycycline; EBDF-quadruple, EPZ + Colloidal bismuth tartrate + Furazolidone + Doxycycline; EBDA-quadruple, EPZ + Colloidal bismuth tartrate + Amoxicillin + Doxycycline.

### Medication adherence analysis

Since some patients in the MITT analysis were lost to follow-up, they could not be surveyed on the MMAS-8 scale, and their tablets could not be counted. Thus, the medication adherence of patients in the MITT analysis was not conducted.

A total of 115 (78.96%), 124 (85.52%), 115 (79.86%), and 124 (85.52%) patients in the VDF-triple, VDA-triple, EBDF-quadruple, and EBDA-quadruple therapy groups, respectively were assessed as having medium medication adherence using the MMAS-8. For tablet counting, most patients took over 90% of the tablets in each group. No differences were observed between the groups (Table 2).

**TABLE 2:** Medication adherence for each group in the PP analysis.

	VDF-triple (N = 144)	VDA-triple (N = 145)	EBDF-quadruple (N = 140)	EBDA-quadruple (N = 133)	*p*-value (VDF-triple VS. VDA-triple)	*p*-value (EBDF-quadruple VS. EBDA-quadruple)	*p*-value (VDF-triple VS. EBDF-quadruple)	*p*-value (VDA-triple VS. EBDA-quadruple)
MMAS-8 score	7.02 ± 0.83	7.21 ± 0.73	7.00 ± 0.82	7.07 ± 0.78	0.053	0.451	0.722	0.121
MMAS-8 assessment	—	—	—	—	0.406	0.785	0.371	0.561
Low	21 (14.58%)	14 (9.66%)	21 (14.58%)	14 (9.66%)	—	—	—	—
Medium	115 (79.86%)	124 (85.52%)	115 (79.86%)	124 (85.52%)	—	—	—	—
High	8 (5.56%)	7 (4.83%)	8 (5.56%)	7 (4.83%)	—	—	—	—
Tablets counting	—	—	—	—	0.980	0.956	0.365	0.335
≥90%	122 (84.72%)	123 (84.83%)	122 (84.72%)	123 (84.83%)	—	—	—	—
<90%	22 (15.28%)	22 (15.17%)	22 (15.28%)	22 (15.17%)	—	—	—	—

Note: VDF-triple, VPZ + Furazolidone + Doxycycline; VDA-triple, VPZ + Amoxicillin + Doxycycline; EBDF-quadruple, EPZ + Colloidal bismuth tartrate + Furazolidone + Doxycycline; EBDA-quadruple, EPZ + Colloidal bismuth tartrate + Amoxicillin + Doxycycline.

### AEs incidence

In the MITT analysis, 38 (23.75%), 39 (24.37%), 46 (28.75%), and 50 (31.25%) patients in the VDF-triple, VDA-triple, EBDF-quadruple, and EBDA-quadruple therapy groups, respectively experienced AEs. No differences were observed between the VDF-triple group vs. the VDA-triple group, EBDF-quadruple group VS. EBDA-quadruple group, VDF-triple group VS. EBDF-quadruple, and VDA-triple group VS. EBDA-quadruple group.

In the PP analysis, the highest incidence of AEs was in the VDA triple group, comprising 26 (18.57%) patients. Most patients experienced moderate AEs, with abdominal pain, diarrhea, and nausea being the most common AEs. The incidence of abdominal pain was significantly higher in the VDF-triple and VDA-triple groups (*p* = 0.009). However, no difference was observed between the patients in the EBDF- and EBDA-quadruple groups. The incidence of diarrhea was significantly higher in the EBDF quadruple group than in the VDF triple (*p* = 0.026) and VDA triple (*p* = 0.047) groups. The incidence of headaches was significantly higher in the EBDF quadruple group than in the VDF-triple group and the EBDA-quadruple than in the VDA triple groups (Table 3).

**TABLE 3:** Adverse event for each group in PP analysis.

	VDF-triple (N = 144)	VDA-triple (N = 145)	EBDF-quadruple (N = 140)	EBDA-quadruple (N = 133)	*p*-value (VDF-triple VS. VDA-triple)	*p*-value (EBDF-quadruple VS. EBDA-quadruple)	*p*-value (VDF-triple VS. EBDF-quadruple)	*p*-value (VDA-triple VS. EBDA-quadruple)
Adverse events	—	—	—	—	0.767	0.783	0.459	0.869
Yes	22 (15.28%)	24 (15.55%)	26 (18.57%)	23 (17.29%)	—	—	—	—
No	122 (84.72%)	121 (83.45%)	114 (81.43%)	110 (82.71%)	—	—	—	—
The severity of adverse events	—	—	—	—	0.667	1.000	1.000	0.666
Mild	20 (90.91%)	20 (83.33%)	24 (92.31%)	21 (91.30%)	—	—	—	—
Moderate	2 (9.09%)	4 (16.67%)	2 (7.69%)	2 (8.70%)	—	—	—	—
Severe	0 (0.00%)	0 (0.00%)	0 (0.00%)	0 (0.00%)	—	—	—	—
Adverse event terms								
Abdominal pain	15 (10.42%)	4 (2.76%)	11 (7.86%)	6 (4.51%)	0.009*	0.253	0.455	0.527
Diarrhea	4 (2.78%)	14 (9.66%)	3 (2.14%)	10 (7.52%)	0.026*	0.047*	1.000	0.526
Nausea	5 (3.47%)	6 (4.14%)	5 (3.57%)	5 (3.76%)	0.767	0.934	0.964	0.872
Rash	1 (0.69%)	2 (1.38%)	1 (0.71%)	1 (0.75%)	1.000	1.000	1.000	1.000
Headache	1 (0.69%)	1 (0.69%)	9 (6.43%)	7 (5.26%)	1.000	0.682	0.010*	0.030*
Total	26	27	29	29	—	—	—	—

Note: VDF-triple, VPZ + Furazolidone + Doxycycline; VDA-triple, VPZ + Amoxicillin + Doxycycline; EBDF-quadruple, EPZ + Colloidal bismuth tartrate + Furazolidone + Doxycycline; EBDA-quadruple, EPZ + Colloidal bismuth tartrate + Amoxicillin + Doxycycline. *p < 0.05.

### Eradication rate

**FIGURE 2:**
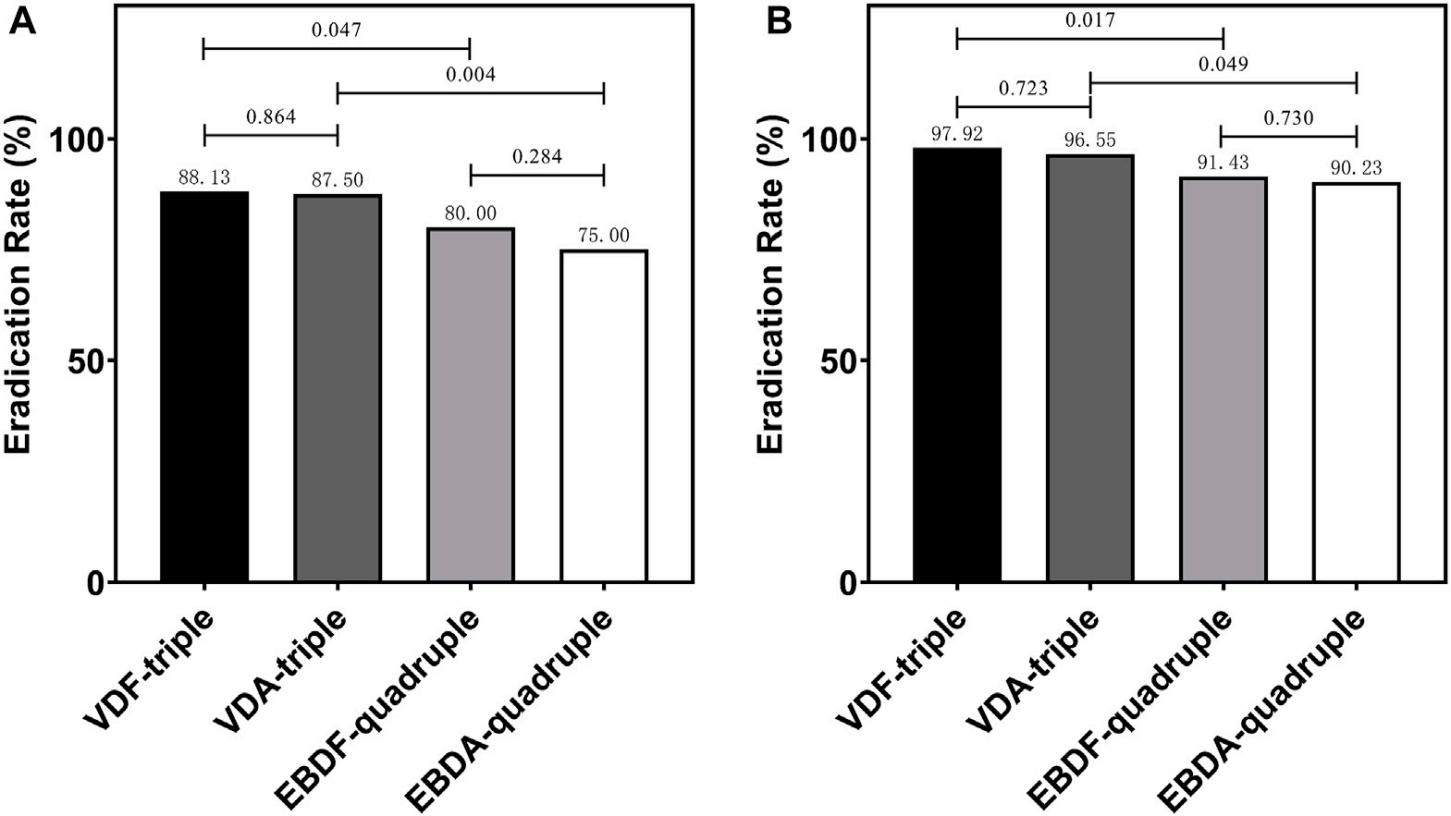
The eradication rate in MITT analysis **(A)** and PP analysis **(B)** VDF-triple group, VPZ + Furazolidone + Doxycycline; VDA-triple group, VPZ + Amoxicillin + Doxycycline; EBDF-quadruple group, EPZ + Colloidal Bismuth Tartrate + Furazolidone + Doxycycline; EBDA-quadruple group, EPZ + Colloidal Bismuth Tartrate + Amoxicillin + Doxycycline.

In the MITT analysis, 141 (88.13%), 140 (87.50%), 128 (80.00%), and 120 (75.00%) patients had *H. pylori* successfully eradicated in the VDF-triple, VDA-triple, EBDF-quadruple, and EBDA-quadruple therapy groups, respectively. There was no significant difference between the groups. The eradication rate was significantly higher in the VDF-triple group than in the EBDF-quadruple and VDA-triple groups. In the PP analysis, the eradication rate was also significantly higher in the VDF-triple group than in the EBDF-quadruple and VDA-triple groups. No differences were observed between the groups (Figure 2). Furthermore, the eradication rate was significantly higher in the VDF-triple + VDA-triple groups (triple therapy) than in the EBDF-quadruple + EBDA-quadruple groups (quadruple therapy), with 87.81% vs. 77.50% in the MITT analysis and 97.23% vs. 90.84% in the PP analysis, respectively.

### Factor analysis

Univariate analysis was conducted in the PP analysis, including patient’s gender, age, gastroscopy result, treatment, whether extra drugs were taken, MMAS-8 score, MMAS-8 assessment, whether patients took ≥90% of drugs (adherence), and whether AEs occurred. We found only treatment, the MMAS-8 score, and adherence correlated with the eradication rate; the Pearson correlation coefficients were 0.134, −0.108, and 0.086, respectively.

Logistic regression analysis was performed using the three correlated variables as covariates and *H. pylori* eradication success as the dependent variable. The final model included two variables: treatment (odds ratio [OR] = 1.720) and MMAS-8 score (OR = 0.610, *p* < 0.001).

### Cost-effectiveness analysis

For each patient who underwent *H. pylori* eradication, the total costs for VDF-triple, VDA-triple, EBDF-quadruple, and EBDA-quadruple groups averaged 702.68 yuan, 711.82 yuan, 829.72 yuan, and 846.78 yuan, respectively. The CER of each group was 7.18 yuan, 7.37 yuan, 9.07 yuan, and 9.38 yuan, respectively. Compared with the VDF-triple group, the ICER for VDA-triple, EBDF-quadruple, and EBDA-quadruple groups were 6.67 yuan, 19.57 yuan, and 18.74 yuan, respectively (Table 4).

**TABLE 4:** The cost-effectiveness analysis in each group.

	VDF-triple	VDA-triple	EBDF-quadruple	EBDA-quadruple
Cost (direct medical costs per patient, yuan) (range)	702.68 (545.08–873.08)	711.82 (546.76–874.76)	829.72 (652.60–980.60)	846.78 (654.28–982.28)
Diagnosis				
Outpatient cost, yuan	10	10	10	10
Gastroscopy cost, yuan	140.93 (0–278)	149.54 (0–278)	162.83 (0–278)	175.58 (0–278)
Testing cost, yuan	116.67 (100–150)	115.52 (100–150)	114.29 (100–150)	116.92 (100–150)
Treatment				
Drugs, yuan	325.08	326.76	432.60	434.28
Follow-up				
Outpatient cost, yuan	10	10	10	10
Testing cost, yuan	100	100	100	100
Eradication rate	97.92	96.55	91.43	90.23
CER, yuan per percent	7.18	7.37	9.07	9.38
ICER, yuan per percent	-	6.67	19.57	18.74

Note: CER, cost-effectiveness ratio; ICER, incremental cost-effectiveness ratio. VDF-triple, VPZ + Furazolidone + Doxycycline; VDA-triple, VPZ + Amoxicillin + Doxycycline; EBDF-quadruple, EPZ + Colloidal bismuth tartrate + Furazolidone + Doxycycline; EBDA-quadruple, EPZ + Colloidal Bismuth Tartrate + Amoxicillin + Doxycycline.

### Antibacterial ability

**FIGURE 3:**
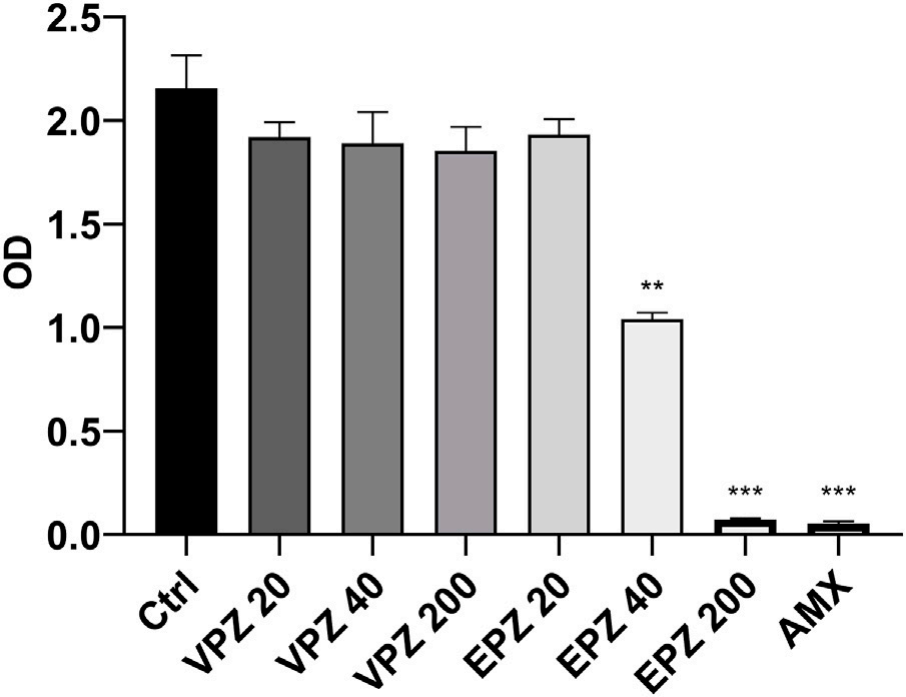
OD values of *H. pylori* at different concentrations. Crtl, control group; VPZ, vonoprazan; EPZ, esomeprazole; AMX, amoxicillin. The units of the post-drug values on the abscissa were μg/ml. **p < 0.01, ***p < 0.001.

The optical density (OD) value of *H. pylori* treated with amoxicillin was significantly lower than that of the negative control (*p* < 0.001), indicating that amoxicillin could significantly inhibit *H. pylori* growth. Compared to the negative control group, EPZ inhibited the growth of *H. pylori*, and the ability to inhibit growth was positively correlated with the concentration of EPZ. The OD value of *H. pylori* in the 200 μg/mL EPZ group was not significantly different from that of the amoxicillin group (*p* = 0.051). The OD values of *H. pylori* at 40 μg/mL and 200 μg/mL EPZ were significantly different from those of the negative control group (*p* = 0.005 and < 0.001, respectively) (Figure 3).

### Transcriptomic result

According to the results of the *in vitro* antibacterial experiments, *H. pylori* cultured in 200 μg/mL VPZ, 40 μg/mL EPZ, and negative control groups were selected for transcriptomic sequencing and analysis. The biological detection and analysis of each sample are shown in Appendix 2.

Compared with the control group, there were 175 differentially expressed genes in *H. pylori* treated with VPZ, of which 96 genes were upregulated, and 79 were downregulated; the most significantly downregulated gene was ptlG (logFC = −1.78), one of the components of the type IV secretion system that mediates the transfer of macromolecules (Sgro et al., 2019).

**FIGURE 4:**
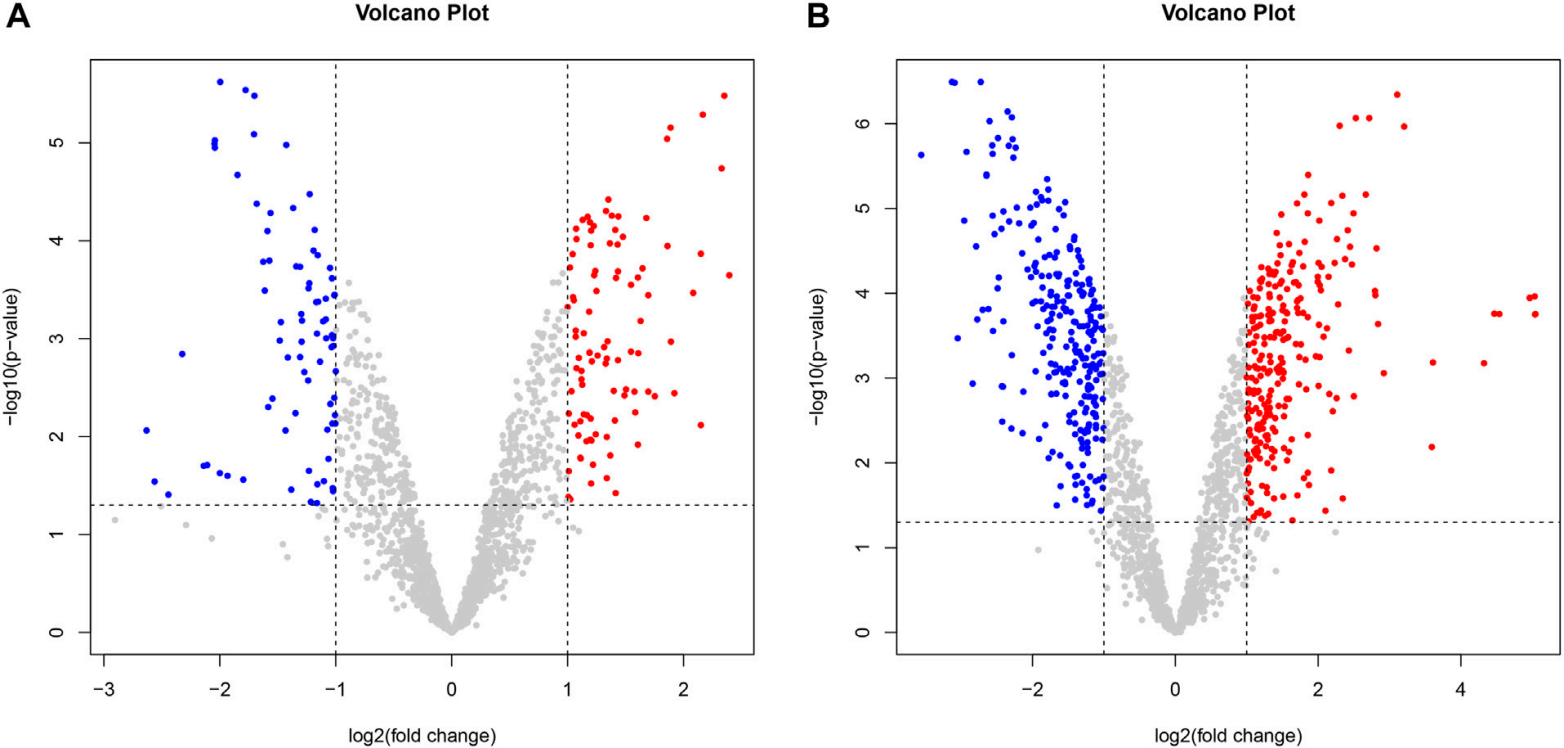
Volcano plot of differentially expressed genes between two groups. Gray dots represented genes that were not differentially expressed, blue dots represented differentially downregulated genes, and red dots represented differentially upregulated genes. **(A)**. VPZ and control groups; **(B)**. EPZ and control groups.

Compared with the control group, *H. pylori* treated with EPZ had 526 differentially expressed genes, of which 263 genes were upregulated and 263 were downregulated; the most significantly downregulated gene was rplL (logFC = −3.13), and its expression product was 50 S ribosomal protein L7/L12, which was used for ribosome synthesis. At the same time, ureB was another differential gene whose expression was significantly downregulated. The logFC value was −2.79. Its product is the beta subunit of urease. Urease is a key protein secreted by *H. pylori*, which allows *it to* adapt to the acidic environment (Kao et al., 2016) (Figure 4).

**FIGURE 5:**
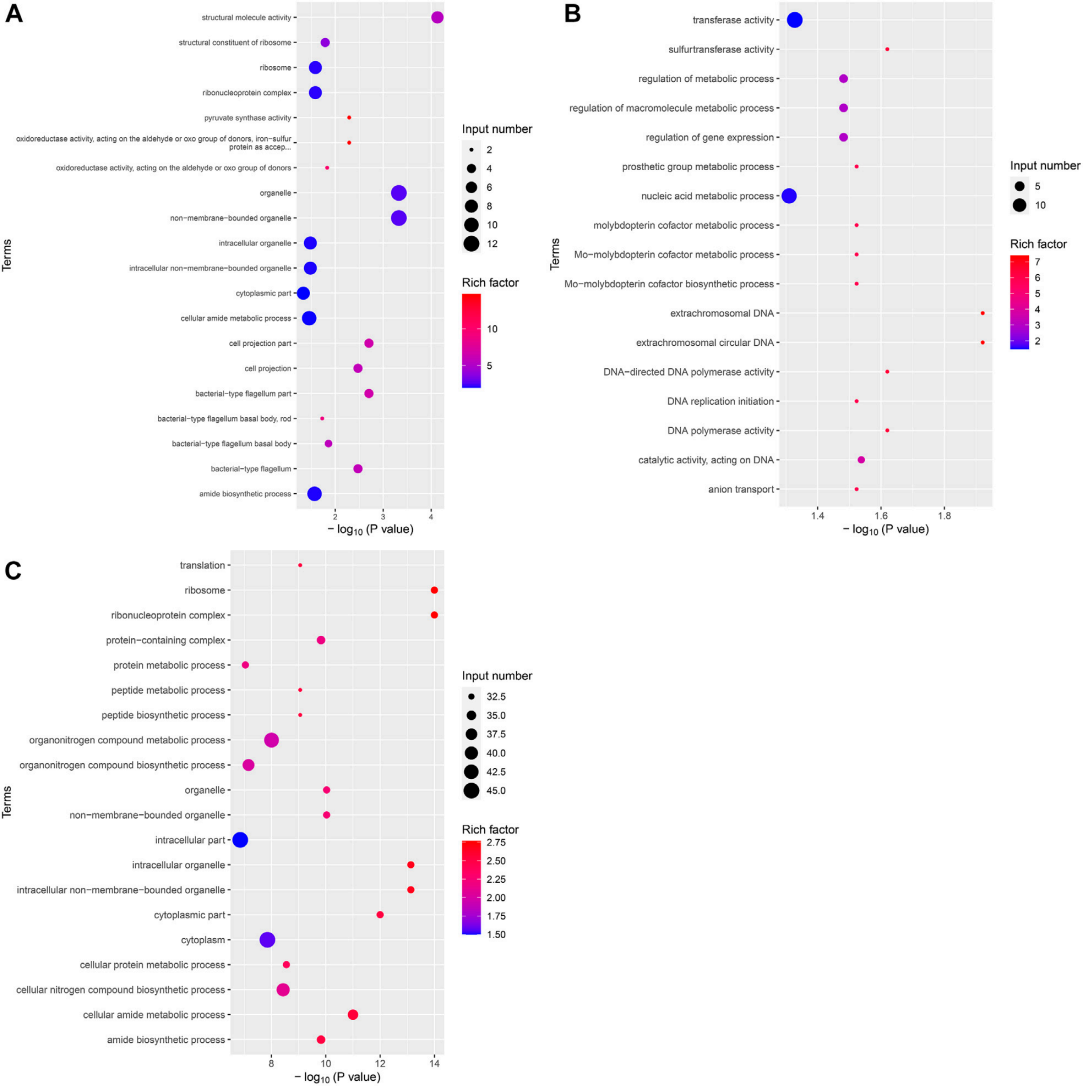
Bubble plot of differentially expressed genes GO enrichment analysis. The horizontal axis represented the significance of enrichment with -log10 (*p*-value); the larger the value, the more significant the enrichment, and the vertical axis represented the enriched GO terms. The size of the dots indicated the number of differential genes contained in the GO terms, and the depth of the dots indicated the degree of enrichment. **(A)** Upregulated differential gene set in VPZ and control groups. **(B)** Upregulated differential gene set in EPZ and control groups. **(C)** Downregulated differential gene set in EPZ and control groups.

After identifying the differentially expressed genes among the groups, GO, and KEGG analyses were performed on these differentially expressed gene sets. Twenty GO terms were enriched in the upregulated differential gene set in the VPZ and control groups. Structural molecule activity was the most significantly enriched GO term, containing seven genes, including flaA and rplC. The downregulated gene set of *H. pylori* treated with VPZ was not significantly enriched for any GO term (Figure 5A). Seventeen GO terms were enriched in the upregulated differential gene set in the EPZ and control groups. Extrachromosomal DNA was the most significantly enriched GO term, including repA. Only plasmids with an intact repA gene could replicate autonomously in *H. pylori*, and no studies have confirmed that the upregulation of this gene could inhibit the activity of *H. pylori* (Heuermann and Haas, 1995) (Figure 5B). Thirty-one GO terms were enriched in the downregulated differential gene set in the EPZ and control groups. Ribosomes were the most significantly enriched GO term. This term contained 33 differential genes related to ribosome syntheses, such as rplA, rplB, and rplL. The proteins expressed by these genes were all subunits of ribosomal proteins, and the lack of these subunits affected the translational function of the ribosome, resulting in *H. pylori* death (Dinos, 2017; Lien et al., 2019) (Figure 5C).

**FIGURE 6:**
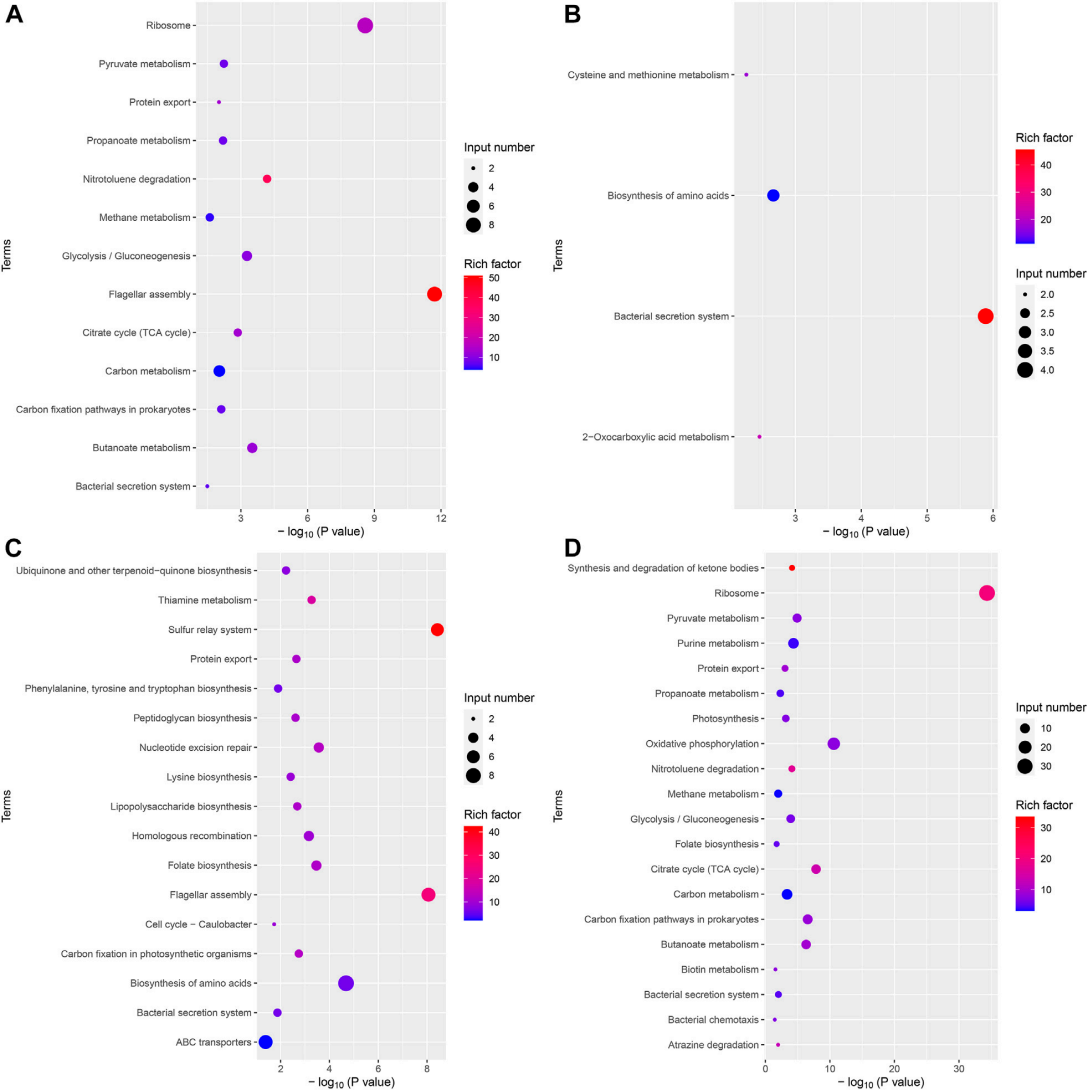
Bubble plot of differentially expressed genes KEGG analysis. The horizontal axis represented the significance of enrichment with -log10 (*p*-value); the larger the value, the more significant the enrichment, and the vertical axis represented the KEGG pathway. The size of the dots indicated the number of differential genes contained in the KEGG pathway, and the depth of the dots indicated the degree of enrichment. **(A)** Upregulated differential gene set in VPZ and control groups. **(B)** Downregulated differential gene set in VPZ and control groups. **(C)** Upregulated differential gene set in EPZ and control groups. **(D)** Downregulated differential gene set in EPZ and control groups.

The results of the KEGG analysis were similar to those of the GO analysis. It should be noted that the bacterial secretion system was a significantly downregulated pathway in the VPZ and control groups. This pathway contained four differentially expressed genes, ptlG and HPYLSS1-00041. HPYLSS1-00041 was found to express a binding transfer protein, but there was no evidence that this protein was involved in the transfer process of *H. pylori* virulence proteins (Figure 6).

### The validation of transcriptomic result

**FIGURE 7:**
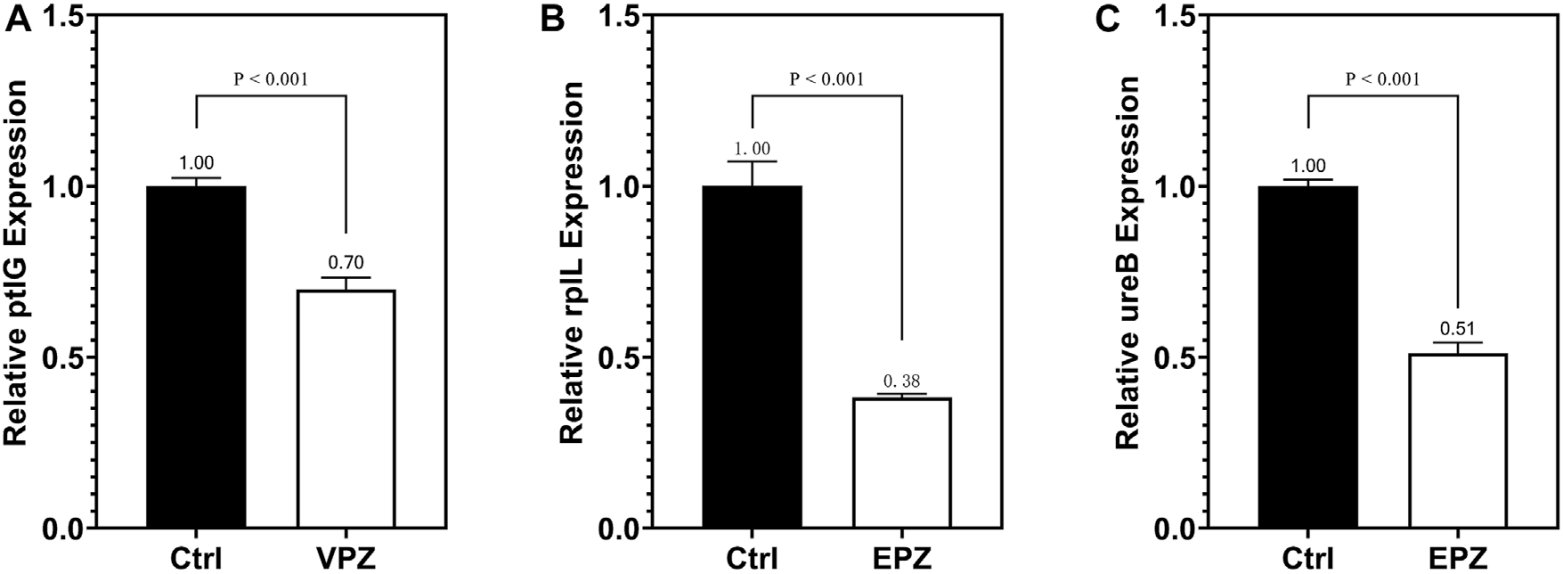
qRT-PCR was used to detect the expression of the ptlG gene in VPZ and control groups **(A)** qRT-PCR detection of rplL gene **(B)** and ureB **(C)** gene expression in EPZ and control groups.

qRT-PCR was performed to detect ptlG. Compared with the control group, we found that the relative expression level of the ptlG gene in the VPZ group was 0.70 (*p* < 0.001), indicating that the ptlG gene was downregulated. When detecting the rplL and ureB genes, compared with the control group, we found that the relative gene expression levels of the prlL and ureB groups in the EPZ group were 0.38 and 0.51, respectively (*p* = 0.001, 0.001), suggesting that rplL and ureB genes were downregulated (Figure 7).

## Discussion

To the best of our knowledge, this is the first study to explore the efficacy and safety of a 14-day VPZ-containing triple therapy in China. In this study, we found that the eradication rate was significantly higher in patients who underwent VPZ-containing triple therapy (87.81% in MITT analysis and 97.23% in PP analysis) than in those who underwent bismuth quadruple therapy, which is highly recommended by the Chinese *H. pylori* eradication guideline (Chinese Society of Gastroenterology et al., 2017) (77.50% in MITT and 90.84% in PP analysis). Regarding safety, the incidence of AEs with triple therapy was not higher than that with quadruple therapy. The CER of triple therapy was lower than that of quadruple therapy. Since the eradication rate is significantly higher in VPZ-containing therapy and the incidence rate of adverse is similar to PPI-containing therapy, traditional PPI can be replaced by VPZ in *H. pylori* eradication in the future. Therefore, we suggest that VPZ-containing triple therapy could be a first-line therapy for *H. pylori* eradication. However, there is no public data on why VPZ-containing treatments can have a higher eradication rate. In this study, we found that the strong acid-suppressing ability of VPZ, rather than its direct inhibitory effect, was key to *H. pylori’s* high eradication rate.

In the previous study, William explored that VPZ 14-day therapy were superior to PPI 14-day therapy (Chey et al., 2022). In their study, bismuth were not used in the PPI 14-day therapy which can significantly increase the eradication rate and commonly used in China (Chinese Society of Gastroenterology et al., 2017). At the same time, the effectiveness of drugs is very different between different races and countries. For example, the mean night-time pH > 4.0 was different in Japanese (100%) and British when taking 40 mg VPZ (Jenkins et al., 2015). Therefore, it is important to conduct a clinical trail to explore the efficiency and safety of the VPZ 14-day therapy. There are different treatments for *H. pylori* eradication, including standard triple therapy (PPI + two kinds of antibiotics), bismuth quadruple therapy (PPI + bismuth + two kinds of antibiotics), and high-dose amoxicillin double treatment. In China, owing to the severe resistance of *H. pylori* to antibiotics, the Chinese guidelines recommend a 14-day treatment to eradicate *H. pylori* (Chinese Society of Gastroenterology et al., 2017). The main factors affecting the *H. pylori* eradication rate include sensitivity to the drugs, especially antibiotic resistance, the effect of acid-suppressing drugs, and medication adherence (Wermeille et al., 2002; McNicholl et al., 2012; Sugimoto and Yamaoka, 2018; Gu and Yang, 2020; Yan et al., 2020). Antibiotic resistance is the main reason for eradication failure; in particular, the abuse of antibiotics significantly increases the resistance of *H. pylori* (Megraud et al., 2013; Megraud et al., 2021). The antibiotic resistance of *H. pylori* in China used in this study was 0%–1% (furazolidone), 0%–5% (amoxicillin), and 0%–5% (doxycycline) (Chinese Society of Gastroenterology et al., 2017; Hu, 2020). We found no significant difference in the eradication rates of different antibiotic combinations when the same basic drugs were used. Therefore, as long as these antibiotics were used, compatible types of antibiotics did not affect the *H. pylori* eradication rate. Besides, clarithromycin was not used in this study since the resistance rate of *H. pylori* to clarithromycin increased rapidly in recent years (Wermeille et al., 2002; McNicholl et al., 2012; Sugimoto and Yamaoka, 2018; Gu and Yang, 2020; Yan et al., 2020). However, some studies have shown that the eradication rate of VPZ-clarithromycin containing therapy is significantly higher than that of clarithromycin without VPZ in other countries (Abadi, 2017; Li et al., 2018; Okubo et al., 2020). In the future, we will conduct new clinical trials to further verify in the Chinese population.

Patient medication adherence is one of the factors affecting the eradication rate of *H. pylori* (Wermeille et al., 2002). Regardless of the treatment regimen used, the eradication rate in patients with high medication adherence was significantly higher than that in patients with low medication adherence (Liou et al., 2016). Although the number of drugs in the VPZ-containing triple therapy was less than that in the bismuth-containing quadruple therapy, we found no significant differences in medication adherence in this study.

Therefore, in this study, the decisive factor for improving the *H. pylori* eradication rate was VPZ vs. PPI + bismuth. Similar to our findings, several studies on VPZ vs. PPI have shown that the VPZ regimen has a higher sterilization rate than the PPI regimen. In a randomized, multicenter, double-blind study of 641 patients, the eradication rate was 92.6% in the 7-day VPZ-containing triple therapy, which was significantly higher than that in the PPI-containing triple therapy (75.9%, *p* < 0.001) (Murakami et al., 2016). Sho et al. also found that the eradication rate of a 7-day VPZ was higher than that of PPI-containing therapy (89.1% vs. 70.9%, *p* < 0.001) (Suzuki et al., 2016). Interestingly, the overall eradication rate in the present study was higher than those reported in previous studies; this may be because the 14-day strategy used in our study prolonged the drug duration of action. It should be pointed out that in this study, we used 14-day therapy instead of 7-day therapy. This is because Chinese guidelines require a 14-day treatment due to more serious antibiotic abuse and differences in prevalent strains in China. Whether the 7-day eradication therapy is equally effective as 14-day is unknown. This study provide the theoretical basis for verifying the effectiveness of the 7-day VPZ-containing therapy which may finally improve Chinese guideline.

In addition, we found that the incidence of abdominal pain in the VDF-triple group was 10.42%, which was higher than that in the VDA-triple group, which was 2.76%. This phenomenon was not observed in the EBDF- and EBDA-quadruple groups. Although abdominal pain is one of the common adverse reactions to furazolidone (Machado et al., 2008), its incidence is higher with VPZ than with EPZ + bismuth, which may be due to the drug interaction between VPZ and furazolidone. In order to further verify this speculation, the further study would be conducted in subsequent clinical practice.

Acid-suppressing drugs are another major factor affecting the eradication rate of *H. pylori* (McNicholl et al., 2012). To avoid the influence of CYP2C19 pharmacogenetic polymorphism, we chose EPZ as one of the drugs in bismuth quadruple therapy (McNicholl et al., 2012; Ke et al., 2021). Ke et al. (2021) found that the pH threshold for *H. pylori* eradication was 5.7. Kagami et al. (Kagami and Furuta, 2016) found that a holding time ratio (HTR) of pH > 6 could reach 85% in patients who received VPZ 20 mg bid. In contrast, the HTR at pH > 6 in the EPZ was only 69%. These data suggest that VPZ has a significantly stronger ability to inhibit gastric acid secretion than EPZ; this is because VPZ inhibits resting and active proton pumps, while PPI only affects active proton pumps (Martinucci et al., 2017). It should be pointed out that 24 h intragastric pH monitoring was not performed in this study because the ability of VPZ to inhibit gastric acid has been well documented.

The exact mechanism for the high eradication rate of VPZ-containing triple therapy is still unknown (Murakami et al., 2016). Since EPZ and omeprazole have been shown to kill *H. pylori in vitro*, we assumed that VPZ might have a stronger bacteriostatic ability than PPI. To clarify this mechanism further, we performed *in vitro* experiments. Unexpectedly, we found that VPZ did not show a direct inhibitory effect on *H. pylori*; the minimum inhibitory concentration (MIC) of VPZ was over 200 μg/mL, while that of EPZ was between 40 μg/mL and 200 μg/mL. The MIC of EPZ was slightly different from that reported by Gatta et al. (2003), which may be related to the fact that they used wild *H. pylori* strains isolated from gastric tissues of patients, whereas we used standard *H. pylori* strains.

Although VPZ does not show the ability to inhibit the growth of *H. pylori in vitro* directly, it may affect other pathways, such as adhesion and fixation, to assist in the eradication of *H. pylori*. Although previous studies have confirmed that EPZ has a direct inhibitory effect on *H. pylori*, the underlying mechanism remains unclear. Therefore, we performed a transcriptomic analysis of *H. pylori* treated with 200 μg/mL VPZ and 40 μg/mL EPZ. We found very few differentially expressed genes in *H. pylori* treated with VPZ compared to the control group. The most downregulated gene was ptlG. Its product is a type IV secretion system component, which can mediate the transfer of macromolecules (Sgro et al., 2019). Current studies indicate that the cytotoxin-related type IV secretion expressed by the CagI, CagY, and CagA genes can lead to the injection of virulence proteins into target cells rather than the ptlG gene (Backert et al., 2017; Lettl et al., 2020). Significantly downregulated genes were not enriched in GO analysis, and only a few pathways were enriched in KEGG analysis, among which the “bacterial secretion system” was the most obvious. Interestingly, pathways related to flagellar assembly were enriched in significantly upregulated enrichment analyses, which indicated that VPZ did not reduce the pathogenicity of *H. pylori* by inhibiting its adhesion and colonization. Therefore, VPZ has neither a direct inhibitory effect on *H. pylori* nor has been shown to reduce the pathogenicity of *H. pylori* through other pathways. For EPZ, genes involved in ribosomes, represented by the rplL, were significantly downregulated. GO and KEGG analyses revealed that terms or pathways related to ribosome synthesis were significantly downregulated. Previous studies have shown that anise lactone and clarithromycin can inhibit the growth of *H. pylori* by inhibiting ribosome synthesis (Dinos, 2017; Lien et al., 2019). Therefore, we believe that EPZ can also inhibit *H. pylori* infection by inhibiting ribosome synthesis.

This study had several limitations. First, this was a single-center study, which biased the clinical study results. We are currently conducting a multicenter study to explore the efficiency and safety of VPZ-containing triple therapy. In addition, our experiments were mainly carried out at the transcriptomic level, and we will carry out further experiments, including protein level detection, in the future.

Summarily, in the Chinese population, the eradication rate of *H. pylori* with the 14-day VPZ-containing triple therapy was significantly higher than that with the 14-day bismuth-containing quadruple therapy. There was no significant difference in the incidence of AEs related to triple and quadruple therapies. The CER of 14-day VPZ-containing triple therapy was lower than that of 14-day bismuth-containing quadruple therapy. EPZ had an antibacterial effect on *H. pylori in vitro* in a concentration-dependent manner. EPZ may have a bacteriostatic effect by inhibiting the ribosome synthesis of *H. pylori*. The high eradication rate of VPZ-containing triple therapy was due to the stronger acid-suppressing ability of VPZ rather than the direct bacteriostatic effect on *H. pylori.*

## Data Availability

The original contributions presented in the study are included in the article/Supplementary Material. The transcriptomic data presented in this study can be found at https://www.ncbi.nlm.nih.gov/sra/PRJNA928378.
