# Supplementary material for: The efficacy and mechanism of vonoprazan-containing triple therapy in the eradication of Helicobacter pylori
Source: Front Pharmacol. 2023 May 5;14:1143969. doi: 10.3389/fphar.2023.1143969 (PMC10196117; doi:10.3389/fphar.2023.1143969)
Supplement: Supplementary file 1 [file DataSheet2.docx]

In terms of sample biological repeat correlation detection, the correlation heat map between samples was shown in Figure SA. The intra-group repeatability of the EPZ group and the control group was very good and there were differences between the groups. The principal component analysis cluster diagram of the gene expression levels of the samples in the VPZ group, the EPZ group and the control group was shown in Figure SB.


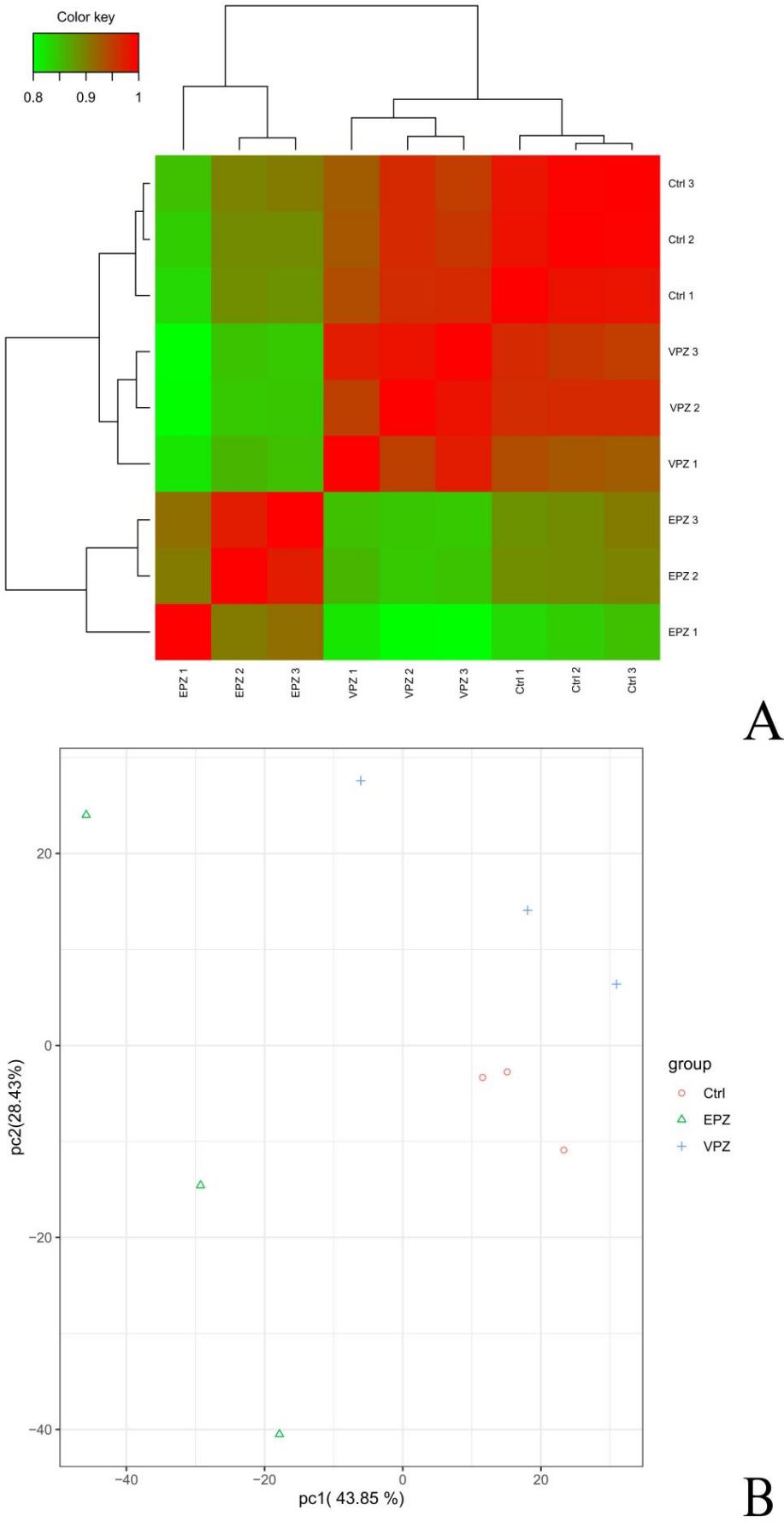


Figure S. Biological detection and analysis of each sample. A. Hierarchical clustering diagram of gene expression levels between samples, the color scale from green to red represents lower and higher inter-sample correlations obtained by gene expression levels, respectively. The branch line segments generated by the same node represent that the corresponding samples can be grouped into one class, and the length of the branch represents the similarity of the samples: the shorter the length, the higher the similarity between samples; the longer the length, the lower the similarity between samples; B. The cluster diagram of the principal component analysis of the gene expression level of the sample, the abscissa represents the first-ranked principal component dimension, and the ordinate represents the second-ranked principal component dimension. VPZ, vonoprazan; Ctrl, control group; EPZ, esomeprazole.
