# Supplementary material for: The efficacy and mechanism of vonoprazan-containing triple therapy in the eradication of Helicobacter pylori
Source: Front Pharmacol. 2023 May 5;14:1143969. doi: 10.3389/fphar.2023.1143969 (PMC10196117; doi:10.3389/fphar.2023.1143969)
Supplement: Supplementary file 2 [file DataSheet1.docx]

Total RNA was extracted from the control, VPZ and EPZ groups using TRIzol reagent (Invitrogen, cat. NO 15596026) following the method of Chomczynski et al. The extracted RNA was DNA digested with DNase I. RNA quality was determined by A260/A280 using a Nanodrop™ OneC Spectrophotometer (Thermo Fisher Scientific Inc). RNA integrity was confirmed by 1.5% agarose gel electrophoresis. Qualified RNA was quantified on Qubit 3.0 using the Qubit™ RNA Broad Range Assay Kit (Life Technologies, Q10210). Qualified RNA was finally quantified by Qubit3.0 and Qubit™ RNA Broad Range Assay Kit (Life Technologies, Q10210).

The stranded RNA sequencing library was built by 2 μg total RNA through Ribo-off rRNA Depletion Kit (Bacteria) (Catalog NO. MRZB12424, Illumina) and KC-DigitalTM Stranded mRNA Library Prep Kit for Illumina sequencing platform (Catalog NO. DR08502, Wuhan Seqhealth Co., Ltd. China). These kits eliminate duplication bias in PCR and sequencing steps by labeling pre-amplified cDNA molecules with a unique molecular identifier (UMI) containing 8 random bases. Library products corresponding to 200-500 bps were enriched, quantified and finally sequenced on the NovaSeq 6000 (Illumina) sequencing platform.

Raw sequencing data was first filtered by Trimmomatic (version 0.36), low-quality reads were discarded and the reads contaminated with adaptor sequences were trimmed. Clean Reads were further treated with in-house scripts to eliminate duplication bias introduced in library preparation and sequencing. In brief, clean reads were first clustered according to the UMI sequences, in which reads with the same UMI sequence were grouped into the same cluster. Reads in the same cluster were compared to each other by pairwise alignment, and then reads with sequence identity over 95% were extracted to a new sub-cluster. After all sub-clusters were generated, multiple sequence alignment was performed to get one consensus sequence for each sub-clusters. After these steps, any errors and biases introduced by PCR amplification or sequencing were eliminated.

Deduplicated Reads were mapped to the reference genome of SS1 strain from NCBI (https://www.ncbi.nlm.nih.gov/nuccore/CP009259.1) using STRA software (version 2.5.3a) with default parameters. Reads mapped to the exon regions of each gene were counted by featureCounts (Subread-1.5.1; Bioconductor) and then RPKM was calculated. Genes differentially expressed between groups were identified using the edgeR package (version 3.12.1). p-value cutoff of 0.05 and Fold-change cutoff of 2 were used to judge the statistical significance of gene expression differences. Gene ontology (GO) analysis and Kyoto encyclopedia of genes and genomes (KEGG) enrichment analysis for differentially expressed genes were both implemented by KOBAS software (version: 2.1.1) with a P-value cutoff of 0.05 to judge statistically significant enrichment. Alternative splicing events were detected by using rMATS (version 3.2.5) with an FDR value cutoff of 0.05 and an absolute value of Δψ of 0.05.
